# Simultaneous Determination of Five Cytochrome P450 Probe Substrates and Their Metabolites and Organic Anion Transporting Polypeptide Probe Substrate in Human Plasma Using Liquid Chromatography-Tandem Mass Spectrometry

**DOI:** 10.3390/pharmaceutics10030079

**Published:** 2018-07-02

**Authors:** Jae-Kyung Heo, Hyun-Ji Kim, Ga-Hyun Lee, Boram Ohk, Sangkyu Lee, Kyung-Sik Song, Im Sook Song, Kwang-Hyeon Liu, Young-Ran Yoon

**Affiliations:** 1 BK21 Plus KNU Multi-Omics based Creative Drug Research Team, College of Pharmacy, Kyungpook National University, Daegu 41566, Korea; anna4602@gmail.com (J.-K.H.); khj110917@nate.com (H.-J.K.); lgh2710@gmail.com (G.-H.L.); sangkyu@knu.ac.kr (S.L.); 2 College of Pharmacy and Research Institute of Pharmaceutical Sciences, Kyungpook National University, Daegu 41566, Korea; kssong@knu.ac.kr (K.-S.S.); isssong@knu.ac.kr (I.S.S.); 3 Clinical Trial Center, Kyungpook National University Hospital, Daegu 41566, Korea; dhrqhfka@naver.com; 4 Department of Biomedical Science, BK21 Plus KNU Bio-Medical Convergence Program for Creative Talent, College of Medicine, Kyungpook National University, Daegu 41944, Korea

**Keywords:** cytochrome P450, drug interaction, liquid chromatography-tandem mass spectrometry, organic anion transporting polypeptide, pharmacokinetics

## Abstract

A rapid and selective liquid chromatography-tandem mass spectrometry (LC-MS/MS) method for the simultaneous determination of organic anion transporting polypeptide 1B1 (OATP1B1) and cytochrome P450 (P450) probe substrates and their phase I metabolites in human plasma was developed. The OATP1B1 (pitavastatin) and five P450 probe substrates, caffeine (CYP1A2), losartan (CYP2C9), omeprazole (CYP2C19), dextromethorphan (CYP2D6), and midazolam (CYP3A) and their metabolites were extracted from human plasma (50 µL) using methanol. Analytes were separated on a C18 column followed by selected reaction monitoring detection using MS/MS. All analytes were separated simultaneously within a 9 min run time. The developed method was fully validated over the expected clinical concentration range for all analytes tested. The intra- and inter-day precisions for all analytes were lower than 11.3% and 8.82%, respectively, and accuracy was 88.5–117.3% and 96.1–109.2%, respectively. The lower limit of quantitation was 0.05 ng/mL for dextromethorphan, dextrorphan, midazolam, and 1′-hydroxymidazolam; 0.5 ng/mL for losartan, EXP-3174, omeprazole, 5′-hydroxyomeprazole, and pitavastatin; and 5 ng/mL for caffeine and paraxanthine. The method was successfully used in a pharmacokinetic study in healthy subjects after oral doses of five P450 and OATP1B1 probes. This analytical method provides a simple, sensitive, and accurate tool for the determination of OATP1B1 and five major P450 activities in vivo drug interaction studies.

## 1. Introduction

Cytochrome P450 (P450) enzymes are responsible for the oxidative metabolism of xenobiotics and endogenous substrates, and are major sources of variability in drug metabolism and pharmacokinetics [1,2]. Currently, 57 different isoforms have been characterized in humans [3]. Among them, five P450 isoforms, CYP1A2, 2C9, 2C19, 2D6, and 3A are involved in the metabolism of more than 90% of marketed drugs [4]. Especially, CYP3A4 and CYP3A5 are major isoforms implicated in the biotransformation of macrolide antibiotics, antihistamines, benzodiazepines, calcium channel blockers, and statins [5]. In addition, CYP1A2, 2C9, 2C19, and 2D6 are implicated in the biotransformation of many drugs (CYP1A2 for caffeine, phenacetin, and tizanidine; CYP2C9 for angiotensin blockers, nonsteroidal anti-inflammatory drugs, and sulfonylureas; CYP2C19 for proton pump inhibitors and antiepileptics; and CYP2D6 for beta blockers, antidepressants, and antipsychotics) [6,7]. The modulation of P450 activities by drug interactions could affect the pharmacokinetics and pharmacodynamics of drugs. In addition to drug metabolizing enzymes, drug transporters can also cause various pharmacological consequences. Drug interactions mediated by permeability-glycoprotein (P-gp) and organic anion transporting polypeptides (OATPs) have been reported for their association with clinically important drug interactions [8]. Recent data have suggested that OATP1B1 is involved in the pharmacokinetics of some protease inhibitors (saquinavir and ritonavir [9]) and statin drugs (pravastatin and pitavastatin [10]).

The inhibition of P450s or OATPs increases plasma levels of the substrate drugs [11], whereas their induction conversely decreases plasma levels of substrate drug. These unwanted drug interactions can result in adverse drug reactions or therapeutic failure. Therefore, accurate and reliable measurements of the in vivo activity of drug-metabolizing enzymes and transporters are essential in evaluating drug interactions. For rapid and efficient evaluation of drug interactions, the cocktail (the simultaneous administration of multiple probe drugs) phenotyping method which provides information on the activities of multiple enzymes or transporters in a single experiment has been widely developed and used. It is important to develop simultaneous analytical methods for probe substrates and their metabolites in cocktail phenotyping studies. To date, there is limited data on the simultaneous analysis of probe substrates of drug metabolizing enzymes and transporters. For example, Kim et al. [12,13] reported a simultaneous analytical method for P-gp (fexofenadine) and five P450 probe substrates (caffeine for CYP1A2, losartan for CYP2C9, omeprazole for CYP2C19, dextromethorphan for CYP2D6, and midazolam for CYP3A) using liquid chromatography-tandem mass spectrometry (LC-MS/MS) in plasma. Bosilkovska et al. [14] also developed a simultaneous LC-MS/MS method for P-gp (fexofenadine) and six P450 probe substrates (caffeine for CYP1A2, bupropion for CYP2B6, flurbiprofen for CYP2C9, omeprazole for CYP2C19, dextromethorphan for CYP2D6, and midazolam for CYP3A) in plasma. However, there is no published data on the simultaneous analysis of OATP and multiple P450 probe substrates and their metabolites in plasma.

Several probe substrates have been validated to assess the activity of P450s and OATP1B1. Among them, caffeine, omeprazole, dextromethorphan, and midazolam are generally the most used as probes for CYP1A2, 2C19, 2D6, and 3A, respectively [15,16,17]. However, several different probe drugs have been used for CYP2C9 and OATP1B1 phenotyping studies. Flurbiprofen [14,18], losartan [15,19], tolbutamide [20], and warfarin [21] have been used for CYP2C9 phenotyping. However, tolbutamide is no longer commercially available in many countries [13], and the data for flurbiprofen and warfarin are not entirely clear [13,15]. OATP1B1 activity has been evaluated using pitavastatin [22], pravastatin [23], and rosuvastatin [24]. OATP1B1 is the most important transporter for the hepatic uptake of pitavastatin [25], while OATP1B1 and sodium-taurocholate cotransporting polypeptide (NTCP) plays an important role in rosuvastatin uptake [26]. OATP1B1 and organic anion transporter 3 (OAT3) are mainly responsible for the transport of pravastatin [27,28]. Therefore, in this study, we selected caffeine, losartan, omeprazole, dextromethorphan, midazolam, and pitavastatin as probe drugs for CYP1A2, 2C9, 2C19, 2D6, 3A, and OATP1B1, respectively. These probe drugs are commercially available for in vivo phenotyping studies, are relatively safe, and are specific for P450 isoforms and OATP1B1.

The present study, for the first time, describes an LC-MS/MS method that was developed to simultaneously analyze five P450-specific probe drugs and their metabolites as well as an OATP1B1 probe drugs in human plasma. The developed method is simpler and faster for small sample volumes than conventional models, and it uses protein precipitation method followed by LC-MS/MS analysis. The method was validated for selectivity, sensitivity, linearity, accuracy, precision, and stability. In addition, the method was successfully used to measure the plasma concentration of the probe drugs and their metabolites in plasma samples from healthy subjects after a single oral dose of the probe drug cocktail, which contained caffeine, losartan, omeprazole, dextromethorphan, midazolam, and pitavastatin.

## 2. Materials and Methods

### 2.1. Chemicals and Reagents

Caffeine, omeprazole, and propranolol were purchased from Sigma-Aldrich (St. Louis, MO, USA). Dextromethorphan, dextrorphan, midazolam, 5′-hydroxyomeprazole, losartan, losartan carboxylic acid (EXP3174), paraxanthine, and pitavastatin were obtained from Toronto Research Chemicals Inc. (North York, ON, Canada). 1′-Hydroxymidazolam was purchased from Cayman Chemical (Ann Arbor, MI, USA). Solvents were LC-MS grade (Fisher Scientific Co., Pittsburgh, PA, USA) and the other chemicals were obtained from Sigma-Aldrich. Pooled human plasma was obtained from BioChemed Services (Winchester, VA, USA).

### 2.2. Preparation of Calibration Standard Samples

**Figure 1:**
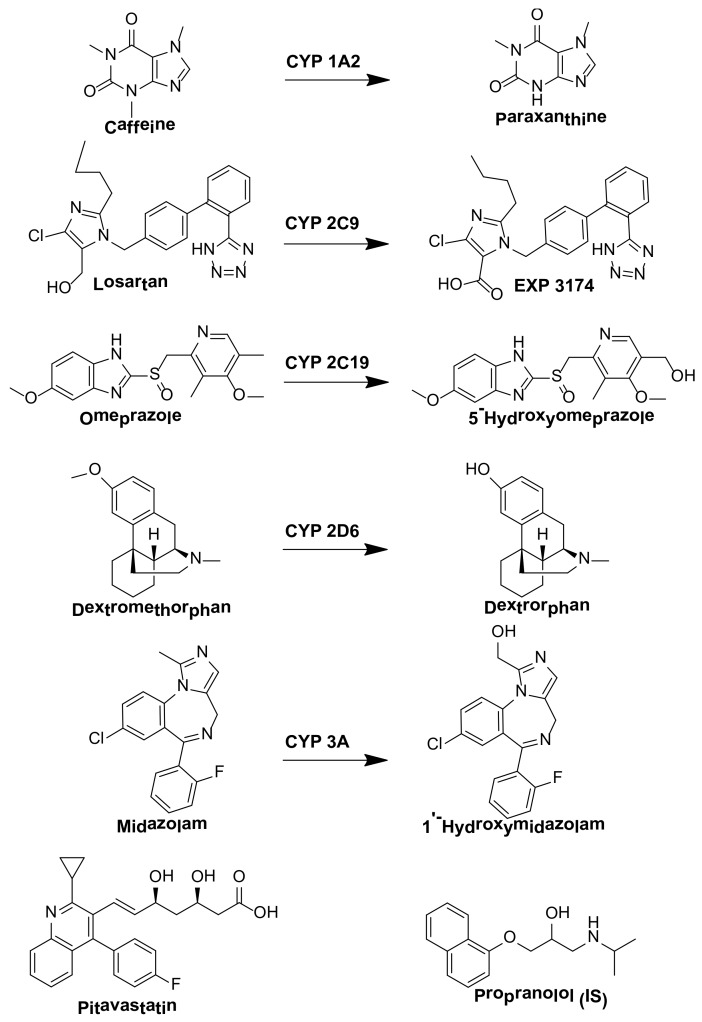
Chemical structure of organic anion transporting polypeptide (OATP) and cytochrome P450 (P450) probe drugs, their metabolites, and propranolol (internal standard [IS]) used in this study.

Stock solutions of the probe substrates, their metabolites, and propranolol (internal standard, IS) were prepared at 1 mg/mL in methanol. Paraxanthine was prepared as a 1 mg/mL solution in 50% aqueous methanol. All stock solutions were sonicated for 5 min. Working standard solutions were prepared by diluting the stock solutions in methanol. All stock and working solutions were stored at −20 °C. Calibration standards were prepared by spiking drug-free blank plasma with the working solutions to obtain concentrations within the relevant analytical ranges (5, 10, 20, 40, 100, 400, 1000, and 4000 ng/mL for caffeine and paraxanthine; 0.5, 1, 2, 4, 10, 40, 100, and 400 ng/mL for losartan, EXP3174, omeprazole, 5′-hydroxyomeprazole and pitavastatin; and 0.05, 0.1, 0.2, 0.4, 1, 4, 10, and 40 ng/mL for dextromethorphan, dextrorphan, midazolam and 1′-hydroxymidazolam) (Table 1 and Figure 1). Calibration curves for the analytes in plasma were constructed from their peak area ratios relative to that the IS using linear regression. All calibration standards samples were stored frozen at −80 °C.

### 2.3. Plasma Sample Preparation

A simple protein precipitation method was used to extract the probe drugs and their metabolites from human plasma. IS solution (10 µL of 5 µg/mL propranolol) and methanol (140 µL) were added to a 50 µL of human plasma sample, which was vortexed for 10 s, and then centrifuged for 15 min (4 °C). The supernatant was transferred to an autosampler vial and 5 µL was injected into the LC-MS/MS system for the analysis.

### 2.4. LC-MS/MS Analysis

The probe drugs and their metabolites were analyzed using a Shimadzu LCMS-8060 1iquid chromatograph-mass spectrometer system (Shimadzu, Tokyo, Japan) equipped with an electrospray ionization (ESI) interface. Analyte separation was performed using the Xbridge MS C18 column (100 × 2.1 mm, i.d., 3.5 µm; Waters, Milford, MA, USA). The mobile phase consisted of 0.1% formic acid in water (A) and 0.1% formic acid in acetonitrile (B), and was run on the following gradient: 0–1 min (5% B), 3–4 min (80% B), and 4.1–9 min (5% B). The flow rate was 0.2 mL/min. The column oven was maintained at a constant temperature of 40 °C. The electrospray ionization was conducted in the positive ion mode at 4000 V. The optimum operating conditions were as follows: vaporizer temperature, 300 °C; capillary temperature, 350 °C; and collision gas (argon) pressure 1.5 mTorr. Quantitation was performed in the selected reaction monitoring (SRM) of the [M + H]^+^ ion and the related product ion for each drugs and its metabolites. The SRM transitions and collision energy (CE) values were determined for the drugs and their metabolites (Table 2).

### 2.5. Method Validation

The developed method was validated for accuracy, linearity, precision, sensitivity, selectivity, and stability for all probe drugs and their metabolites. The selectivity was tested by analyzing human plasma samples from six different sources. The linearity of the calibration curve was examined using eight calibration points for each analyte with different concentration (Table 1). Least squares regression was used to construct the calibration curve for each analyte. To evaluate the linearity, the acceptable criteria were set at ±15% deviation of the nominal concentrations except at the lower limit of quantitation (LLOQ, ±20%), which was defined as the concentrations of the signal-to-noise ratio at 10. Quality control (QC) samples were prepared at final concentration of 20, 100, and 1000 ng/mL for caffeine and paraxanthine; 2, 10, and 100 ng/mL for losartan, EXP3174, omeprazole, 5′-hydroxyomeprazole and pitavastatin; and 0.2, 1, and 10 ng/mL for dextromethorphan, dextrorphan, midazolam, and 1′-hydroxymidazolam. The precision and accuracy were assessed by analyzing QC samples at three different concentration levels (low, middle, and high) with five replicates within one day and on six consecutive days for the intra- and inter-day validation, respectively. The precision was defined as the relative standard deviation (RSD, %), and the accuracy was calculated as follows: (mean observed concentration)/(nominal concentration) × 100. The acceptable criteria were set at ±15% deviation of the nominal concentration. The storage stability of the analytes was determined using triplicate spiked samples after 4 h at room temperature. In addition, the freeze–thaw stability of the analytes was assessed for three freeze-thaw cycles. The acceptable criteria for stability test were within a 15% loss of the initial concentrations.

### 2.6. Application to Pharmacokinetic Studies

Six healthy male volunteers who provided written informed consent participated in the pharmacokinetic study, which was approved (No. 2017-01-010) by the Institutional Review Board of Kyungpook National University Hospital (Daegu, Korea) and performed according to the guidelines of good clinical practice. After an overnight fast, all subjects received a single oral dose of the probe drug cocktail, which contained caffeine (100 mg), losartan (50 mg), omeprazole (20 mg), dextromethorphan (30 mg), midazolam (2 mg), and pitavastatin (2 mg). Blood samples were collected into a tube containing ethylenediaminetetraacetic acid before (0 h) and at 0.25, 0.5, 0.75, 1, 1.5, 2, 3, 4, 5, 6, 8, 10, 12, 24, and 48 h after cocktail administration. Following centrifugation at 1811× *g* for 10 min, the supernatant plasma was stored at −70 °C until the analysis. The following pharmacokinetic parameters were obtained using non-compartmental methods with Phoenix WinNonlin 7.0 (Pharsight Corporation, Certara, NJ, USA): the maximum plasma concentration (C_max_), time to reach C_max_ (T_max_), area under the plasma concentration-time profile (AUC), the half-life (t_1/2_) in the terminal phase, and mean residence time (MRT).

## 3. Results and Discussion

### 3.1. Optimization of Analytical Conditions and Sample Preparation

To develop a reliable LC-MS/MS method suitable for the simultaneous detection of all the probe drugs and their metabolites, chromatographic and spectrometric conditions such as mobile phase, column, SRM transition ions, and collision energies were optimized. The mobile phase used had an acetonitrile content that differed slightly from that used in a previously reported method [13]. All analytes spiked into the plasma samples at 0.05–5 ng/mL, were sensitively detected and eluted within 4 min using gradient elution of 0.1% formic acid in water and acetonitrile (Table 1). For sensitive and selective analysis of the target analytes using the SRM mode, mass fragmentation patterns were investigated to select the SRM transition ions at various CE values. All analytes generated a protonated molecular ion [M + H]^+^ in the positive ion mode. Based on the product ion scan mass spectra, the most abundant ions were selected as product ions for quantification (Table 2). All analytes were selectively separated based on retention times within 2.7 to 4 min (Figure 1).

Several sample pretreatment methods, e.g., liquid–liquid extraction [13], solid-phase extraction (SPE) [30], and on-line SPE [31] have been reported for extracting P450 probe drugs and their metabolites in plasma samples. However, previously reported extraction methods required tedious or complex extraction procedures such as double liquid-liquid extraction [13] and hybrid SPE-precipitation [30]. The protein precipitation method we established using methanol was a simple way to extract the 11 target analytes (OATP1B1 and P450 probe drugs and their metabolites) from the human plasma samples. Bosilkovska et al. [14] and Tanaka et al. [32] reported a protein precipitation method using acetonitrile; however, their method did not include pitavastatin (OATP1B1 probe drug) as the target analyte. In addition, previously reported protein precipitation methods requires large volumes (0.3 mL) of plasma [32,33]. The protein precipitation method we established using small volumes (50 µL) of plasma is a simple way to extract the eleven target analytes (OATP1B1 and P450 probe drugs and their metabolites) from the human plasma samples. Although stable isotope-labeled IS samples (such as midazolam-d4 or omeprazole-d3) are the first choices [14,34], they are relatively expensive. Therefore, we investigated several compounds including chlorpropamide [35], paracetamol [31], and terfenadine [36] to find a suitable IS, and finally chose propranolol for use in this assay.

### 3.2. Method Validation

Several research studies have reported the analytical method for five P450 isoform-probe drugs and their metabolites in plasma samples using LC-MS/MS. Recently, Oh et al. [13], Tanaka et al. [32], Williams et al. [16], and Zhang et al. [31] developed an LC-MS/MS method for five P450 probe drugs (caffeine, losartan, omeprazole, dextromethorphan, and midazolam) and their metabolites, which were used in an Inje cocktail [15]. Kim et al. [12] developed a simultaneous assay for four P450 probe drugs (losartan, omeprazole, dextromethorphan, and midazolam) and their metabolites as well as fexofenadine, a P-gp substrate, after protein precipitation; however, caffeine and paraxanthine were separately analyzed after liquid-liquid extraction. Bosilkovska et al. [14] also reported a simultaneous analytical method for five P450 probe drugs (including caffeine, bupropion, flurbiprofen, omeprazole, dextromethorphan, and midazolam) and their metabolites as well as fexofenadine, P-gp substrate. To date, however, there is no report for the simultaneous analysis of five P450 and OATP1B1 probe drugs. In this study, for the first time, we developed an LC-MS/MS method to simultaneously analyze five P450s specific probe drugs (caffeine, losartan, omeprazole, dextromethorphan, and midazolam) and their metabolites as well as an OATP1B1 probe drug (pitavastatin) using LC-MS/MS after protein precipitation in human plasma. The developed method was validated for selectivity, sensitivity, linearity, accuracy, precision, and stability as follows.

**Figure 2:**
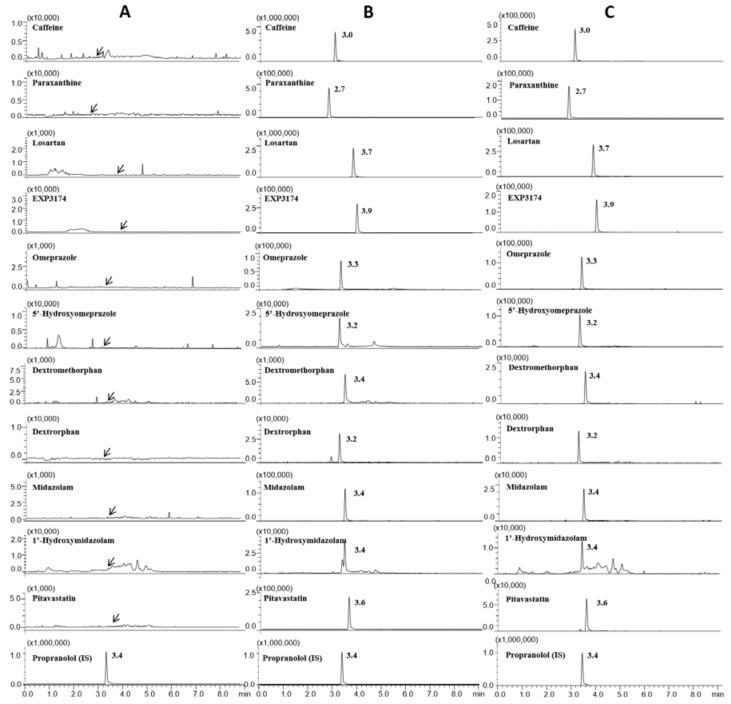
Selected reaction monitoring chromatograms of probe drugs, their metabolites, and internal standard (IS) in (**A**) blank plasma samples spiked with IS, (**B**) plasma samples collected from a subject 1 h after dosing and (**C**) plasma samples spiked with middle quality control (QC) concentrations.

The selectivity of the assay was investigated by preparing and analyzing six independent blank (drug-free) samples. During the experimentation, no significant interfering peaks were observed at the retention times and SRM mass transition for all analytes. Representative SRM chromatograms for plasma samples spiked with the QC samples (100 ng/mL for caffeine and paraxanthine, 10 ng/mL for losartan, EXP3174, omeprazole, 5′-hydroxyomeprazole, and pitavastatin, and 1.0 ng/mL for dextromethorphan, dextrorphan, midazolam, and 1′-hydroxymidazolam) of all analytes and plasma collected from one subject 1 h after dosing are shown in Figure 2. For all analytes, the calibration curves were linear at 5–4000 ng/mL for caffeine and paraxanthine, 0.5–400 ng/mL for losartan, EXP3174, omeprazole, 5′-hydroxyomeprazole, and pitavastatin, and 0.05–40 ng/mL for dextromethorphan, dextrorphan, midazolam, and 1′-hydroxymidazolam (Table 1). These concentration ranges covered the expected plasma concentration for each drug after oral administration of the drug cocktail described above. A weighting factor of 1/(concentration)^2^ was applied to calibration curves for all drugs and their metabolites because of their wide calibration range. There were no interfering peaks in the blank human plasma. The coefficients of correlation (*r*^2^) values were >0.994 for all analytes in all batches in the validation and pharmacokinetic analysis. The RSD values of the correlation coefficients were less than 0.6%. No significant differences in linear regressions were observed among the inter- and intra-day assays, indicating that the assay was reproducible [37]. The LLOQ values were 0.05 ng/mL for dextromethorphan, dextrorphan, midazolam, and 1′-hydroxymidazolam, 0.5 ng/mL for losartan, EXP3174, omeprazole, 5′-hydroxyomeprazole, and pitavastatin, and 5 ng/mL for caffeine and paraxanthine (Table 1). These LLOQ concentrations were chosen based on previously reported values for the administered doses, and they encompassed the expected concentration values of each analyte in plasma.

As shown in Table 3, the accuracy for each analyte and concentration evaluated was in the range of 88.5–114.5%, except for the middle dextromethorphan QC, which was slightly overestimated (117.3%). The intra- and inter-day assay precisions for all analytes were <11.3% and <8.8%, respectively (Table 3), suggesting that the assay had high accuracy and reliability. The data obtained satisfied the pre-defined acceptance criteria for accuracy and precision [13,14].

All analytes in plasma were stable for up to 4 h at 25 °C (Table 4). No degradation, defined as any deviation outside ±15% of the nominal concentration [13], was observed after three freeze–thaw cycles or post-treatment storage for 24 h at 4 °C (Table 4). No differences in stability occurred between the low- and high-concentration QC samples. The analytical procedure was determined to be reliable based on selectivity, sensitivity, linearity, accuracy, precision, and stability and thus, this method was applied to the plasma samples collected from subjects in the pharmacokinetic study.

### 3.3. Clinical Applications

**Figure 3:**
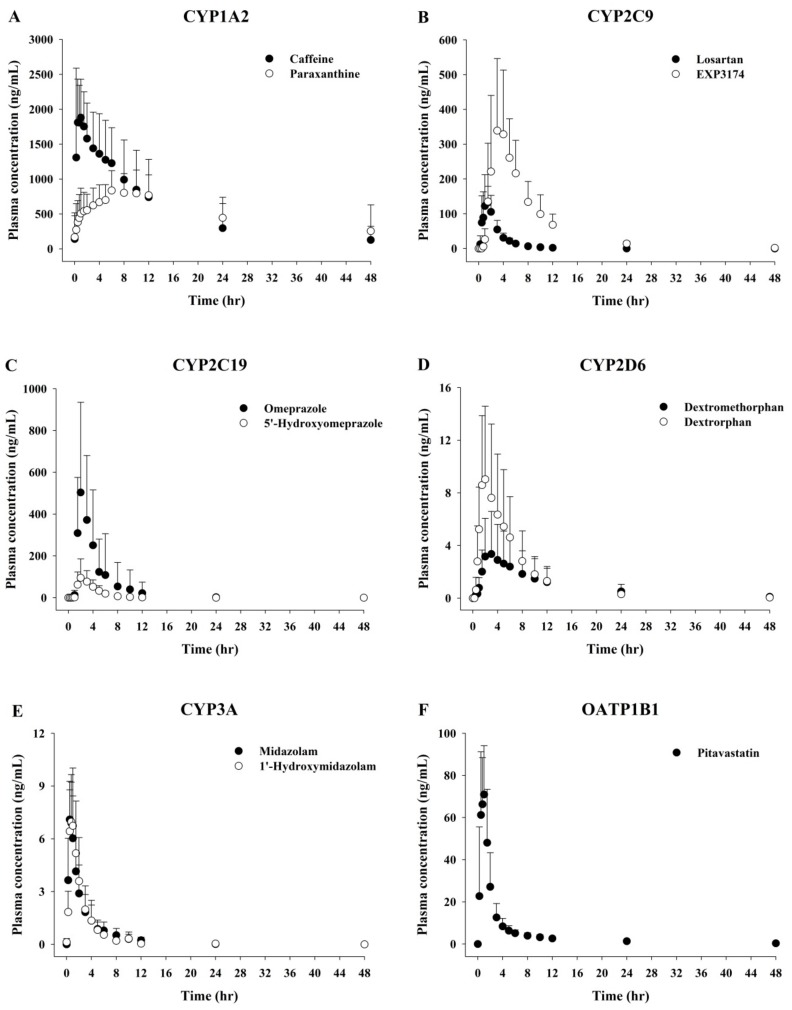
Mean plasma concentration-time profiles for organic anion transporting polypeptide 1B1 (OATP1B1) and cytochrome P450 probe drugs and their metabolites after administration of cocktail drugs (*n* = 6).

These analytical methods were successfully used to determine concentrations of the all the probe drugs and their metabolites in human plasma samples after a single oral dose of the probe drug cocktail in six healthy volunteers. Figure 3 shows the mean plasma concentration–time profiles for OATP1B1 and five P450 probe drugs and their metabolites after the administration of cocktail drugs (Figure 3A,F). Caffeine/paraxanthine, EXP3174, dextromethorphan/dextrorphan and pitavastatin were detected over 48 h. Losartan was detected within 2 h with C_max_ and AUC from time zero to 48 h (AUC_0–48_), values of 172.50 ng/mL and 387.50 h·ng/mL, respectively. Omeprazole and 5′-hydroxyomeprazole were detected within 4 h with C_max_, values of 566.07 and 114.09 ng/mL, respectively. Midazolam was absorbed rapidly resulting in T_max_ of 0.5 h and C_max_ value of 7.61 ng/mL (Table 5). Therefore, the developed analytical method was sufficiently sensitive and selective for use in pharmacokinetic studies.

## 4. Conclusions

In this study, we have developed and validated a rapid, reliable, precise, and selective assay to determine the concentrations of five P450 isoforms (CYP1A2, CYP2C9, CYP2C19, CYP2D6, and CYP3A) probe drugs and their metabolites and the transporter OATP1B1 probe drug in human plasma using protein precipitation followed by a single LC-MS/MS run. The method was also successfully applied to a pharmacokinetic study in healthy subjects who received a cocktail of OATP1B1 and five P450 probe drugs. This method would be useful for the clinical evaluation of P450 and OATP1B1 activity, and in vivo drug–drug interactions of potential of drug candidates.

## Figures and Tables

**Table 1:** Calibration range, linearity, and limit of quantitation (LOQ) of analytes.

Analyte	Retention Time (min)	Calibration Range (ng/mL)	Correlation Coefficient (*r*^2^)	LOQ (ng/mL)
Caffeine	2.9	5–4000	0.9982 ± 0.002	5.0
Paraxanthine	2.7	5–4000	0.9989 ± 0.001	5.0
Losartan	3.7	0.5–400	0.9989 ± 0.001	0.5
EXP3174	3.8	0.5–400	0.9978 ± 0.002	0.5
Omeprazole	3.2	0.5–400	0.9973 ± 0.002	0.5
5′-Hydroxyomeprazole	3.2	0.5–400	0.9984 ± 0.002	0.5
Dextromethorphan	3.4	0.05–40	0.9982 ± 0.002	0.05
Dextrorphan	3.1	0.05–40	0.9979 ± 0.002	0.05
Midazolam	3.4	0.05–40	0.9985 ± 0.001	0.05
1′-Hydroxymidazolam	3.4	0.05–100	0.9938 ± 0.006	0.05
Pitavastatin	3.5	0.5–400	0.9987 ± 0.001	0.5

**Table 2:** Selected reaction monitoring (SRM) transition ion and collision energy (CE) values for the analysis of analytes and internal standard (IS).

Analyte	SRM Transition Ions (*m*/*z*) [13,29]		CE (eV)
	Precursor Ion	Product Ion	
Caffeine	195.0	138.0	20
Paraxanthine	181.0	124.0	20
Losartan	423.0	207.0	23
EXP3174	437.0	235.0	20
Omeprazole	346.0	198.0	15
5′-Hydroxyomeprazole	362.0	214.0	15
Dextromethorphan	272.0	171.0	37
Dextrorphan	258.0	157.0	40
Midazolam	326.0	291.0	27
1′-Hydroxymidazolam	342.0	324.0	23
Pitavastatin	422.0	290.0	33
Propranolol (IS)	261.0	184.0	20

**Table 3:** Intra- and inter-day precision and accuracy of quality control (QC) samples for all probe drugs and their metabolites in human plasma.

Analyte	Nominal Concentration (ng/mL)	Intra-Day (*n* = 5)			Inter-Day (*n* = 6)		
		Measured (ng/mL) *	RSD ** (%)	Accuracy (%)	Measured (ng/mL) *	RSD ** (%)	Accuracy (%)
Caffeine	20.0	22.2 ± 0.4	2.0	110.7	21.8 ± 0.8	3.5	109.2
	100.0	110.2 ± 2.2	2.0	110.2	103.6 ± 4.0	3.9	103.6
	1000	1071.0 ± 26.5	2.5	107.1	975.8 ± 28.0	2.8	97.6
Paraxanthine	20.0	22.2 ± 0.5	2.0	110.9	21.5 ± 0.8	3.7	107.3
	100.0	113.9 ± 0.8	0.7	113.9	102.6 ± 4.2	4.1	102.6
	1000	1073.8 ± 25.8	2.4	107.4	997.1 ± 23.9	2.4	99.7
Losartan	2.0	2.1 ± 0.0	1.5	104.8	2.1 ± 0.1	5.5	103.0
	10.0	10.7 ± 0.2	1.8	107.1	10.0 ± 0.2	1.8	100.4
	100	102.2 ± 0.8	0.8	102.2	99.0 ± 1.9	1.9	99.0
EXP3174	2.0	2.1 ± 0.0	1.9	106.1	2.0 ± 0.1	4.0	101.1
	10.0	10.4 ± 0.2	1.7	104.3	10.0 ± 0.3	3.0	100.0
	100	99.1 ± 1.2	1.5	99.1	98.9 ± 4.6	4.7	98.9
Omeprazole	2.0	2.1 ± 0.1	3.3	104.8	2.0 ± 0.1	5.6	102.0
	10.0	11.2 ± 0.2	1.3	111.8	10.1 ± 0.7	6.5	101.4
	100	106.5 ± 1.0	1.0	106.5	96.7 ± 6.6	6.8	96.7
5′-Hydroxyomeprazole	2.0	2.0 ± 0.1	4.3	101.6	2.0 ± 0.1	2.7	102.0
	10.0	10.7 ± 0.2	1.8	106.6	10.1 ± 0.4	3.6	100.6
	100	101.2 ± 1.3	1.3	101.2	96.1 ± 3.1	3.2	96.1
Dextromethorphan	0.2	0.2 ± 0.0	7.3	97.1	0.2 ± 0.0	5.0	100.0
	1.0	1.2 ± 0.0	2.2	117.3	1.0 ± 0.0	3.1	101.8
	10	11.1 ± 0.1	1.0	111.3	10.2 ± 0.1	1.1	101.7
Dextrorphan	0.2	0.2 ± 0.0	11.3	100.2	0.2 ± 0.0	8.8	103.0
	1.0	1.1 ± 0.0	3.3	106.5	1.0 ± 0.0	2.6	103.4
	10	10.7 ± 0.3	2.5	106.5	10.2 ± 0.1	1.3	101.6
Midazolam	0.2	0.2 ± 0.0	6.3	103.1	0.2 ± 0.0	2.7	102.0
	1.0	1.1 ± 0.0	3.5	111.1	1.0 ± 0.0	1.6	100.8
	10	11.0 ± 0.1	0.6	109.9	10.1 ± 0.1	0.7	100.7
1′-Hydroxymidazolam	0.2	0.2 ± 0.0	6.3	88.5	0.1 ± 0.0	4.1	101.0
	1.0	0.9 ± 0.0	3.8	95.3	1.0 ± 0.1	6.3	99.0
	10	10.7 ± 0.1	1.2	106.9	10.0 ± 0.4	3.5	100.3
Pitavastatin	2.0	2.1 ± 0.1	2.8	105.3	2.1 ± 0.1	4.4	104.9
	10.0	11.5 ± 0.3	2.9	114.5	10.5 ± 0.5	5.1	105.3
	100	107.5 ± 1.6	1.5	107.5	99.2 ± 6.2	6.2	99.2

* Results are expressed as concentration mean ± SD. ** RSD, relative standard deviation.

**Table 4:** Short-term (4 h), freeze–thaw (three cycles), and post-treatment (4 °C, 24 h) stability results for all probe drugs and their metabolites in human plasma. Results are expressed as concentration mean ± SD.

Analyte	Nominal Concentration (ng/mL)	4 h Short-Term Stability (25 °C)	Freeze-Thaw Stability (−80 °C/Room Temperature)	24 h Post-Treatment Stability (4 °C)
Caffeine	100	91.8 ± 2.1	99.4 ± 3.3	96.2 ± 0.8
	1000	96.7 ± 7.6	93.9 ± 5.4	91.7 ± 2.3
Paraxanthine	100	86.1 ± 4.6	94.7 ± 1.4	96.8 ± 4.1
	1000	91.7 ± 7.5	90.0 ± 4.6	92.8 ± 1.5
Losartan	10	94.5 ± 2.1	101.9 ± 2.7	98.4 ± 3.4
	100	93.8 ± 3.8	95.6 ± 3.3	95.0 ± 2.0
EXP3174	10	90.5 ± 2.9	95.2 ± 2.6	98.8 ± 1.4
	100	91.9 ± 6.3	92.9 ± 4.0	93.1 ± 1.9
Omeprazole	10	89.8 ± 1.2	93.8 ± 1.8	94.7 ± 2.0
	100	88.9 ± 6.5	88.4 ± 3.5	94.6 ± 1.6
5′-Hydroxyomeprazole	10	99.0 ± 3.7	99.9 ± 6.6	96.5 ± 2.2
	100	91.0 ± 4.1	91.1 ± 4.8	91.0 ± 1.1
Dextromethorphan	1	107.2 ± 5.3	102.6 ± 6.4	102.5 ± 2.4
	10	100.5 ± 3.1	114.5 ± 3.6	102.5 ± 1.3
Dextrorphan	1	88.3 ± 5.2	91.0 ± 2.0	95.4 ± 12.4
	10	87.4 ± 6.3	92.1 ± 4.9	94.5 ± 4.3
Midazolam	1	105.9 ± 1.3	106.8 ± 5.3	100.5 ± 3.1
	10	104.1 ± 3.4	112.3 ± 1.3	103.6 ± 1.1
1′-Hydroxymidazolam	1	102.0 ± 5.0	99.7 ± 5.9	110.5 ± 0.3
	10	99.9 ± 7.5	107.3 ± 4.8	102.5 ± 3.0
Pitavastatin	10	91.0 ± 2.4	93.5 ± 3.6	99.1 ± 1.7
	100	93.4 ± 9.4	89.7 ± 2.6	95.4 ± 1.2

**Table 5:** Summary of pharmacokinetic parameters of organic anion transporting polypeptide 1B1 (OATP1B1) and cytochrome P450 (P450) probe drugs and their metabolites (*n* = 6). Results are expressed as mean ± SD or median (range).

Probe Drug	Pharmacokinetic Parameters	Mean ± SD
Caffeine	AUC_0–48_ (h·ng/mL)	27,327.6 ± 18,012.4
(CYP1A2)	C_max_ (ng/mL)	2350.4 ± 843.1
	T_max_ (h)	0.75 (0.25–1.50)
	t_1/2_ (h)	8.84 ± 3.01
	MRT (h)	11.1 ± 4.0
Paraxanthine	AUC_0–48_ (h·ng/mL)	24,063.5 ± 13,009.8
	C_max_ (ng/mL)	925.3 ± 305.5
	T_max_ (h)	7 (5–24)
	t_1/2_ (h)	12.46 ± 6.03
	MRT (h)	16.8 ± 4.7
Losartan	AUC_0–48_ (h·ng/mL)	387.5 ± 121.2
(CYP2C9)	C_max_ (ng/mL)	172.5 ± 62.6
	T_max_ (h)	1.25 (0.50–2.00)
	t_1/2_ (h)	2.14 ± 0.53
	MRT (h)	2.7 ± 0.3
EXP3174	AUC_0–48_ (h·ng/mL)	2721.6 ± 1236.2
	C_max_ (ng/mL)	352.1 ± 205.6
	T_max_ (h)	3.5 (3.0–5.0)
	t_1/2_ (h)	6.79 ± 0.58
	MRT (h)	8.6 ± 1.2
Omeprazole	AUC_0–48_ (h·ng/mL)	1796.2 ± 2076.3
(CYP2C19)	C_max_ (ng/mL)	566.1 ± 367.1
	T_max_ (h)	2.0 (1.5–3.0)
	t_1/2_ (h)	1.34 ± 1.14
	MRT (h)	3.5 ± 1.5
5′-Hydroxyomeprazole	AUC_0–48_ (h·ng/mL)	315.6 ± 195.3
	C_max_ (ng/mL)	114.1 ± 80.9
	T_max_ (h)	2.0 (1.5–4.0)
	t_1/2_ (h)	1.59 ± 1.12
	MRT (h)	3.8 ± 1.0
Dextromethorphan	AUC_0–48_ (h·ng/mL)	41.36 ± 40.33
(CYP2D6)	C_max_ (ng/mL)	3.70 ± 3.23
	T_max_ (h)	2.5 (1.0–3.0)
	t_1/2_ (h)	8.50 ± 2.49
	MRT (h)	8.7 ± 4.3
Dextrorphan	AUC_0–48_ (h·ng/mL)	63.91 ± 44.51
	C_max_ (ng/mL)	10.48 ± 5.32
	T_max_ (h)	1.5 (1.0–3.0)
	t_1/2_ (h)	6.44 ± 2.74
	MRT (h)	7.4 ± 1.9
Midazolam	AUC_0–48_ (h·ng/mL)	18.60 ± 9.65
(CYP3A)	C_max_ (ng/mL)	7.61 ± 2.26
	T_max_ (h)	0.5 (0.5–1.0)
	t_1/2_ (h)	3.08 ± 1.43
	MRT (h)	2.8 ± 0.9
1′-Hydroxymidazolam	AUC_0–48_ (h·ng/mL)	17.65 ± 9.87
	C_max_ (ng/mL)	7.77 ± 2.77
	T_max_ (h)	0.75 (0.50–1.00)
	t_1/2_ (h)	3.32 ± 3.30
	MRT (h)	2.5 ± 0.9
Pitavastatin	AUC_0–48_ (h·ng/mL)	198.1 ± 68.4
(OATP1B1)	C_max_ (ng/mL)	81.31 ± 26.04
	T_max_ (h)	0.75 (0.50–1.50)
	t_1/2_ (h)	13.48 ± 5.24
	MRT (h)	9.8 ± 3.4
